# Relationship Between Emotional Eating and Adherence to the Mediterranean Diet Based on Body Weight in University Students and Individuals from Their Social Environment

**DOI:** 10.3390/nu18020256

**Published:** 2026-01-14

**Authors:** Claudia Di Rosa, Chiara Spiezia, Ludovica Di Francesco, Alessandro Guerrini, Fabiola Diadema, Yeganeh Manon Khazrai

**Affiliations:** 1 Research Unit of Food Science and Human Nutrition, Department of Sciences and Technologies for Sustainable Development and One Health, Università Campus Bio-Medico di Roma, Via Alvaro del Portillo, 21, 00128 Roma, Italy; 2 IRCCS Fondazione Don Carlo Gnocchi, 50143 Florence, Italy

**Keywords:** emotional eating, Mediterranean diet (Med Diet), Emotional Eaters Questionnaire (EEQ), MEDI-LITE questionnaire, Mediterranean diet adherence

## Abstract

**Background/Objectives:** Emotional eating refers to the tendency to eat in response to emotions rather than physiological hunger and has been associated with changes in food choices and difficulties in dietary self-regulation. Whether emotional eating influences adherence to the Mediterranean diet remains unclear. This study aimed to examine the association between emotional eating and adherence to the Mediterranean diet. **Methods:** In this cross-sectional study, 245 adults completed the Emotional Eater Questionnaire (EEQ) and the MEDI-LITE questionnaire to assess adherence to the Mediterranean diet. Participants were classified into three emotional eating categories (NO EE, LEE, EE) and stratified by BMI (normal weight vs. overweight). **Results:** Higher EEQ scores were associated with greater disinhibition, stronger food preferences, and a higher sense of guilt in both BMI categories. However, adherence to the Mediterranean diet did not differ significantly across emotional eating groups, and overall MEDI-LITE scores were low in the entire sample. Correlations between emotional eating subscales and specific food groups were weak and did not show a consistent pattern. **Conclusions:** Emotional eating was associated with psychological and behavioral aspects of eating but was not related to adherence to the Mediterranean diet in this population. The uniformly low adherence to the Mediterranean diet may have attenuated potential associations. Further studies using more detailed dietary assessment tools and longitudinal designs are needed to clarify how emotional eating influences food choices over time.

## 1. Background

Eating behavior is the result of the interaction between physiological mechanisms that regulate hunger and satiety and psychological factors that determine food preferences and responses to emotional states [1,2,3]. Emotional eating, defined as the tendency to eat in response to emotions such as stress, anxiety, boredom or sadness, has been associated with increased consumption of highly palatable foods and difficulties in self-regulating food intake [4]. If persistent, this behavior can contribute to unhealthy eating patterns, weight gain, and psychological distress [5]. In particular, emotional eating has been linked to increased reward sensitivity and altered responses within the neurobiological systems involved in stress and hedonic eating, suggesting that emotional signals may prevail over homeostatic signals in vulnerable individuals [6]. These mechanisms underscore the complexity of emotional eating and its potential to interfere with long-term dietary goals, especially in environments where high-energy foods are readily available.

The Mediterranean diet is widely recognized for its health benefits, including a reduced risk of cardiometabolic diseases, improved weight management and increased well-being [7,8,9]. This dietary pattern favors high consumption of vegetables, fruit, legumes, whole grains, fish and extra virgin olive oil, while limiting consumption of red meat and processed foods [10]. Despite its well-established advantages, adherence to the Mediterranean diet has declined in recent years, even in Mediterranean countries [11,12], partly due to changes in lifestyle and the increased availability of ultra-processed foods [13,14].

Since the Mediterranean diet is characterized by stable, long-term eating habits rather than episodic eating behaviors [10], its relationship with emotion-driven eating patterns remains particularly relevant from a public health perspective. Understanding whether emotional eating interferes with adherence to such eating patterns may help clarify why people struggle to maintain healthy eating patterns despite being aware of their benefits. Although emotional eating has been linked to altered food choices, increased snacking, and a preference for high-energy foods [4,15], its relationship with adherence to structured eating patterns such as the Mediterranean diet remains unclear. Existing studies often differ in assessment tools and sample characteristics [16], making it difficult to compare results. Furthermore, few studies have examined whether this relationship varies according to body weight [5]. In addition, most of the available evidence focuses on discrete food categories or specific eating episodes, while few studies have explored how emotional eating may influence overall dietary indices that reflect habitual intake. Tools such as the MEDI-LITE score [17] allow for a standardized assessment of adherence to the Mediterranean diet, but it is unknown whether emotional eating has a significant impact on these broader dietary patterns.

This study aimed to evaluate the association between emotional eating and adherence to the Mediterranean diet in a sample of normal weight and overweight adults, using validated questionnaires (the Emotional Eater Questionnaire [18] and the MEDI-LITE score [17]). We hypothesized that higher levels of emotional eating would be associated with lower adherence to the Mediterranean diet, particularly among individuals with overweight. In addition, we explored whether specific emotional eating dimensions, including disinhibition, food preferences, and guilt, were differentially associated with individual components of the Mediterranean dietary pattern. Given the exploratory nature of the study, no a priori assumptions were made regarding the strength of these associations.

## 2. Materials and Methods

### 2.1. Study Design and Participants

This cross-sectional observational study was conducted between December 2023 and June 2024. The design, conduct, and reporting of this cross-sectional study were guided by the STROBE statement for observational studies.

Data were collected via an anonymous online survey distributed on social media (Instagram and Facebook). The invitation was addressed to university students at the Campus Bio-Medico of the University of Rome and their family members, allowing for the inclusion of individuals of different age groups with a common cultural background.

Participants were recruited using a convenience sampling strategy, primarily involving university students and their family members, and participation was voluntary and unpaid.

Eligibility criteria included:

- Age ≥ 18 years;
- BMI ≥ 18.5 kg/m^2^;
- Provision of informed consent;
- Complete responses to both questionnaires.

Participants who refused consent, were under 18 years of age, had a BMI < 18.5 kg/m^2^ or submitted incomplete questionnaires were excluded.

The survey was disseminated through the university’s social media channels and informal networks; therefore, it was not possible to calculate an accurate response rate. Basic data quality checks (including completeness and internal consistency) were carried out, but specific attention checks were not included.

Given the use of an open online survey, several precautions were adopted to enhance data quality and minimize non-genuine responses. The survey was anonymous but technically configured to allow only one submission per device/account, thereby preventing multiple entries by the same individual. Access to the questionnaire was granted only after electronic informed consent was provided. Only fully completed questionnaires were included in the analyses, and responses were screened for internal consistency and plausibility. Although formal attention-check items were not included, no duplicate or clearly inconsistent response patterns were identified during data screening. Nonetheless, as with all voluntary online surveys, some degree of self-selection bias cannot be excluded and was considered when interpreting the results.

This recruitment approach limits the generalizability of the findings and may have contributed to the relatively homogeneous characteristics of the study population.

### 2.2. Ethics

The study was approved by the Ethics Committee of the Campus Bio-Medico University of Rome (protocol 60.23 OSS, 17 May 2023). All participants provided electronic informed consent before accessing the survey.

### 2.3. Questionnaires

#### Emotional Eating

Emotional eating was assessed using the Emotional Eating Questionnaire (EEQ), validated by Garaulet et al. [18], a validated 10-item instrument designed to assess the extent to which emotions influence eating behavior.

The items are rated on a 4-point Likert scale (0 = never, 3 = always). The EEQ comprises three subscales:

- **Disinhibition** (6 items): loss of control over food consumption in emotional contexts;
- **Food Preferences** (2 items): preference for specific palatable foods;
- **Sense of Guilt** (2 items): negative emotions associated with eating.

The total score ranges from 0 to 30. Based on the cut-off values established by the original validation study, participants were classified as:

- 0–5 = Non-Emotional Eaters (NO EE);
- 6–10 = Low Emotional Eaters (LEE);
- 11–20 = Emotional Eaters (EE);
- 21–30 = Very Emotional Eaters (VEE).

Due to the small sample size of the VEE group, this category was combined with EE for statistical analyses.

This decision was methodologically necessary due to the small size of the VEE subgroup, in order to ensure sufficient statistical power and stability of the analyses; however, it limits the ability to characterize individuals with the highest levels of emotional eating and should be considered when interpreting the results.

### 2.4. Mediterranean Diet Adherence

Adherence to the Mediterranean diet was assessed using the MEDI-LITE score, validated by Sofi et al. [17], to evaluate the habitual consumption of nine food groups characteristic of the Mediterranean dietary pattern.

Each component contributes 0–2 points based on frequency of consumption, with a total score ranging from 0 to 18. Higher scores indicate greater adherence, and values ≥ 8.5 reflect good adherence, as proposed in the original validation.

The nine components include:

- Fruit;
- Vegetables;
- Legumes;
- Cereals;
- Fish;
- Meat and processed meat;
- Dairy products;
- Alcohol;
- Extra-virgin olive oil.

The questionnaire assesses habitual consumption rather than episodic consumption and is not designed to detect specific episodes of emotional eating.

This characteristic may limit its sensitivity in capturing emotion-driven variations in food consumption.

### 2.5. Statistical Analysis

Analyses were conducted using GraphPad Prism 10.4. Since the data did not meet the assumptions of normality, non-parametric tests were applied. Differences between emotional eating categories within each BMI group were assessed using Kruskal–Wallis tests with Dunn’s post hoc analyses. Spearman correlations were used to examine associations between EEQ scores, subscales, MEDI-LITE score, and individual food components.

Effect sizes (ε^2^) were calculated for nonparametric comparisons between groups (0.01 = small, 0.06 = medium, 0.14 = large). Statistical significance was set at *p* < 0.05. No a priori calculation of statistical power was conducted due to the exploratory nature of the study. Given the exploratory design, the sample size, and the limited variability in Mediterranean diet adherence, multivariate regression analyses were not conducted. Therefore, the statistical analyses were intended to be descriptive, and the results should be interpreted with caution.

## 3. Results

### 3.1. Study Population

**Figure 1:**
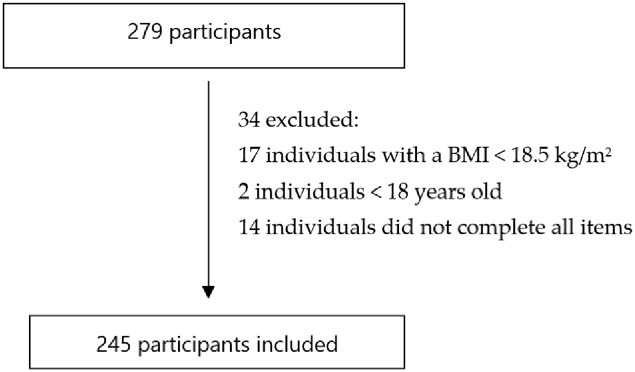
Flow chart of participant selection. A total of 279 participants were assessed for eligibility; 34 were excluded, resulting in a final sample of 245 participants.

A total of 279 individuals completed the online survey. After applying the eligibility criteria, 34 participants were excluded: 1 refused consent, 2 were under 18 years of age, 14 submitted incomplete questionnaires, and 17 had a BMI < 18.5 kg/m^2^. The final analytical sample therefore consisted of 245 adults (Figure 1).

Of these, 160 participants were of normal weight and 85 were overweight. Overweight individuals were on average older and reported a higher prevalence of chronic diseases than participants of normal weight. Anthropometric data are shown in Table 1.

Overall, 80% of respondents reported no chronic diseases. The prevalence of self-reported conditions in the total sample and in each BMI group is shown in Table 2. Gastrointestinal disorders, dyslipidemia, and cardiovascular disease were the most frequently reported conditions in both groups, with the latter two being particularly prevalent among overweight participants.

### 3.2. Questionnaires

(a) Emotional Eating Questionnaire

Based on EEQ total scores, the sample was distributed in categories as follows:

- 18% Non-Emotional Eaters (NO EE),
- 38.7% Low Emotional Eaters (LEE),
- 39.6% Emotional Eaters (EE),
- 3.7% Very Emotional Eaters (VEE).

Due to the small size of the VEE category (*n* = 9), it was merged with the EE category for all analyses. Emotional eating was more common among overweight participants (54%) than among normal weight participants (41%).

The analyses therefore continued with three categories: NO EE, LEE and EE (including VEE). These differences are reported for descriptive purposes only and were not formally tested for statistical significance.

In the normal weight group (*n* = 160), 22% (*n* = 35) were classified as NO EE, 37% (*n* = 59) as LEE and 41% (*n* = 66) as EE; in the overweight group (*n* = 85), the distribution was 11% (*n* = 9), 35% (*n* = 30) and 54% (*n* = 46), respectively.

The mean total EEQ scores for each category are shown in Table 3.

(b) EEQ subscales

Across all BMI groups, EE participants consistently scored higher than both NO EE and LEE participants on all EEQ subscales.

- **Disinhibition:** EE participants scored highest in both weight groups (all *p* < 0.0001). Among normal-weight individuals, LEE also scored higher than NO EE. The size effect for disinhibition was large (ε^2^ = 0.78).
- **Food preferences:** EE participants reported more intense food preferences than both LEE and NO EE participants across all BMI groups.
- **Guilt:** EE participants scored higher on guilt than NO EE in both weight groups, with significantly higher guilt observed in overweight individuals than in normal weight individuals with similar levels of emotional eating. The effect was significant (ε^2^ = 0.80). Overall, the subscale results show a clear gradient in emotional eating characteristics from NO EE to EE.

**Figure 2:**
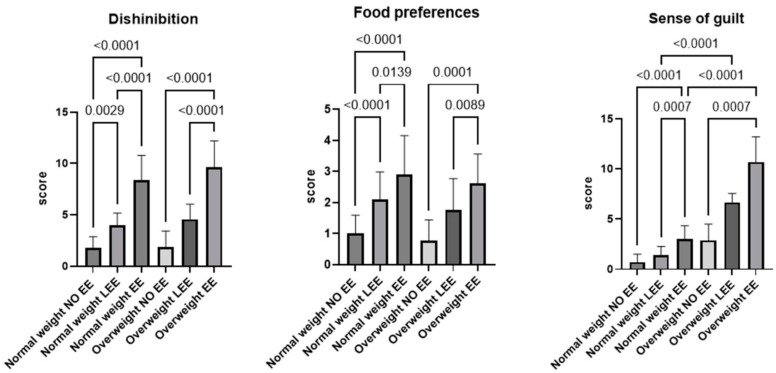
Emotional Eater Questionnaire (EEQ) subscale scores by emotional eating category and body mass index (BMI). Disinhibition, food preferences, and sense of guilt scores are shown for normal-weight and overweight participants classified as non-emotional eaters (NO EE), low emotional eaters (LEE), and emotional eaters (EE). Data are presented as mean ± SD. Group differences were assessed using the Kruskal–Wallis test with Dunn’s post hoc comparisons; *p*-values are reported above the brackets. Analyses were conducted using GraphPad Prism 10.4.

Figure 2 and Table 4 summarize comparisons of the three EEQ subscales.

(c) Mediterranean diet Adherence

Adherence to the Mediterranean diet was low in all categories. Average MEDI-LITE scores ranged between 6 and 7, with no significant differences between emotional eating categories or BMI groups. Approximately 20% of participants scored ≥ 8.5, corresponding to good adherence, with similar proportions across emotional eating levels. No statistically significant differences were found for any of the nine MEDI-LITE components when comparing the NO EE, LEE, and EE categories within each BMI group (Table 5).

(d) Correlations

Correlations between emotional eating indicators and dietary variables were generally weak and inconsistent.

- Among normal-weight participants, small associations emerged:
  ○ In the NO EE category, higher EEQ scores were modestly associated with lower fruit and fish consumption.
  ○ In the LEE category, guilt showed weak positive correlations with fruit consumption and inverse correlations with olive oil consumption.
  ○ In the EE category, weak positive correlations emerged between EEQ scores and vegetable or cereal consumption, and between guilt and several plant-based foods.
- Among participants with overweight, correlations were limited:
  ○ In the NO EE category, some strong associations with fish consumption were observed but these should be interpreted cautiously due to sample size.
  ○ In the LEE category, disinhibition was inversely correlated with cereal consumption.
  ○ In the EE category, no significant correlations were observed.

Overall, the correlation models did not reveal a consistent relationship between emotional eating and adherence to the Mediterranean diet.

The correlation results (with correlation coefficient and *p* values) are reported in Table 6 and Table 7.

## 4. Discussion

In this study, we explored the relationship between emotional eating and adherence to the Mediterranean diet in normal-weight and overweight adults. As expected, higher emotional eating scores were associated with greater disinhibition, stronger food preferences, and higher guilt, regardless of BMI group. These findings are consistent with previous evidence showing that emotional eating is linked to difficulties in regulating eating behavior and greater reactivity to emotional stimuli [4,5,16,19,20,21,22,23,24,25,26,27].

One important factor that may have contributed to the absence of significant associations is the low variability of MEDI-LITE scores within the sample. Adherence to the Mediterranean diet was uniformly low among participants, which likely reduced the ability to detect potential gradients associated with different levels of emotional eating. This limited variability, together with the characteristics of the study population, should be considered when interpreting the present findings.

Emotional eating was also more common among overweight participants, a finding that may reflect the influence of psychological distress and weight-related stigma documented in the previous literature [28,29]. Despite these behavioral differences, adherence to the Mediterranean diet did not vary according to emotional eating levels or weight groups, and overall MEDI-LITE scores were low for the entire sample. This suggests that emotional eating does not necessarily translate into measurable differences in eating habits when assessed using a broad dietary index, particularly in populations where adherence to the Mediterranean diet is uniformly limited [30,31]. Similar trends have been reported in recent studies showing a decline in adherence to traditional Mediterranean eating habits, even in Mediterranean countries [11,12], along with an increase in the consumption of ultra-processed foods [13,32,33,34,35].

Correlational analyses showed only weak and inconsistent associations between emotional eating subscales and specific food groups. Although some relationships reached statistical significance, their magnitude was limited and no consistent pattern emerged. Accordingly, these associations should be interpreted with caution and considered primarily descriptive. These findings may indicate that emotional eating influences episodic or situation-specific food choices (e.g., preference for palatable foods during negative emotional states) [3,4,6,19,21,22,36], which are not easily detected by instruments that assess habitual intake. This highlights a temporal and methodological mismatch between the instruments used: emotional eating is an episodic and context-dependent behavior, whereas the Mediterranean diet reflects long-term eating habits. The instruments used in this study (EEQ and MEDI-LITE) therefore assess distinct temporal dimensions, potentially limiting the identification of associations between emotional states and overall diet quality. Methodological approaches capable of capturing real-time emotional states and food choices may be better suited to investigate this relationship. Future studies could benefit from methodologies such as ecological momentary assessment (EMA) or daily food diaries, which allow for a more sensitive assessment of emotion-driven variations in food choices. Recent ecological momentary assessment (EMA) studies have investigated real-time associations between momentary emotional states and eating behavior, capturing dynamic emotion–intake linkages that are missed by retrospective instruments. For example, in clinical and non-clinical samples, EMA data showed systematic patterns of emotional states preceding binge episodes [37,38], and real-time monitoring approaches have been used to evaluate physical and eating behaviors alongside affective states [39]. A recent meta-analysis further confirmed that negative affect consistently precedes binge eating in EMA studies [40].

Overall, the results suggest that emotional eating primarily reflects psychological and behavioral aspects of eating rather than clear differences in daily eating habits within this population [5,15,22,23,24,25,41]. Future studies using more detailed dietary assessment methods, longitudinal designs, or ecological momentary assessments may help clarify the extent to which emotional eating influences food choices over time [42,43]. From a clinical and research perspective, these findings suggest that emotional eating and overall diet quality should be considered related but distinct dimensions of eating behavior. While emotional eating appears to be strongly associated with psychological and behavioral dysregulation, it may not necessarily translate into poorer adherence to healthy dietary patterns when assessed through broad dietary indices. For clinicians, this highlights the importance of addressing emotional regulation and eating-related distress independently of dietary counseling alone. For researchers, our results underscore the need for integrating dietary assessment tools with methodologies capable of capturing situational and emotion-driven eating episodes, particularly when investigating the relationship between emotional eating and dietary patterns. Differences observed across studies may be partly explained by heterogeneity in study populations, assessment instruments, and analytical approaches, reinforcing the importance of methodological consistency in future research.

## 5. Limitations

This study has several limitations. Dietary intake and anthropometric measurements were self-reported, which may have introduced inaccuracies. The MEDI-LITE questionnaire, although validated, may not detect specific foods typically involved in emotional eating episodes. Furthermore, the cross-sectional design does not allow for causal or temporal inferences.

The sample was recruited through social media and consisted mainly of university students and their relatives; this convenience sampling strategy limits generalizability and may have contributed to the reduced variability of the dietary patterns observed. This relative homogeneity of the sample represents a relevant methodological limitation and may have reduced the ability to detect associations between emotional eating and adherence to the Mediterranean diet. Moreover, potential confounding variables such as age, sex, education level, and socioeconomic status were not included in the analyses and may have influenced the observed associations. Finally, voluntary participation in an open online survey may have introduced self-selection bias, further affecting the observed relationships and attenuating the ability to detect associations between emotional eating and dietary adherence. Additionally, the overall narrow distribution of MEDI-LITE scores may have further limited the ability to detect associations between emotional eating and adherence to the Mediterranean diet. Another limitation concerns the absence of a priori statistical power analysis. Although the study was exploratory in nature, the lack of a formal assessment of statistical power reduces confidence in the ability to detect small but potentially significant effects. Furthermore, the very emotional eating (VEE) subgroup included very few participants, necessitating its merging with the EE category. Although justified from a methodological standpoint, this approach reduces the ability to characterize individuals with the highest levels of emotional eating; larger samples are needed to better understand this subgroup.

## 6. Conclusions

In this sample of normal-weight and overweight adults, emotional eating was associated with greater disinhibition, stronger food preferences, and greater guilt, but was not related to adherence to the Mediterranean diet, as measured by the MEDI-LITE score. Adherence to the Mediterranean diet was generally low in all subgroups, which may have limited the ability to detect potential associations, suggesting that factors other than emotional eating may play a predominant role in shaping daily eating habits in this population.

In this exploratory study, the findings underscore the importance of assessing the emotional and behavioral dimensions of eating independently of habitual eating patterns; however, given the cross-sectional design, the characteristics of the study population, and the limitations of the dietary assessment tool used, the results should be interpreted with caution. Future research using longitudinal designs and more detailed dietary assessment tools such as food diaries or ecological momentary assessment could help clarify how emotional eating influences food choices over time and whether specific emotion-driven eating episodes contribute to long-term changes in diet quality.

## Figures and Tables

**Table 1:** Anthropometric characteristics of the study population. Age, body weight, and height are reported for the total sample and stratified by body mass index (BMI). Data are presented as mean ± SD.

Characteristics	Total Sample (*n* = 245)	Normal Weight (*n* = 160)	Overweight (*n* = 85)
**Age (years) (mean ± SD)**	36.1 ± 14.43	33.5 ± 13.06	40.9 ± 15.64
**Body weight (kg) (mean ± SD)**	68.97 ± 14.03	62.07 ± 8.6	82.04 ± 12.93
**Height (m) (mean ± SD)**	1.69 ± 0.09	1.69 ± 0.09	1.70 ± 0.09

**Table 2:** Self-reported pathological conditions in the study population. The prevalence of reported conditions is shown as percentages for the total sample and stratified by body mass index (BMI).

Condition Category	Total Sample (*n* = 49/245; 20%)	Normal Weight (*n* = 27/160; 17%)	Overweight (*n* = 22/85; 26%)
**Gastrointestinal disorders (IBS, IBD, hiatal hernia, gastroesophageal reflux) (%)**	5.1	5.6	3.4
**Dyslipidemia (%)**	4.5	3.8	6.1
**Cardiovascular diseases (heart disease, Raynaud’s phenomenon or hypertension) (%)**	4	1.3	9.5
**Thyroid problems**	1.6	1.3	2.3
**Allergies or gluten sensitivity**	1.2	0.6	2.3
**Hyperinsulinemia or type 2 diabetes mellitus**	0.8	1.3	-
**Multiple sclerosis or demyelinating diseases**	0.8	1.3	-
**Epilepsy**	0.4	0.6	-
**Type 1 diabetes mellitus**	0.4	-	1.2
**Polycystic ovary syndrome**	0.4	0.6	-
**Sarcoidosis**	0.4	-	1.2
**Cancer**	0.4	0.6	-

**Table 3:** Emotional Eater Questionnaire (EEQ) total scores by emotional eating category and body mass index (BMI). Mean EEQ total scores are reported for normal-weight and overweight participants classified as non-emotional eaters (NO EE), low emotional eaters (LEE), and emotional eaters (EE). Data are presented as mean ± SD.

	Normal Weight Participants			Participants with Overweight		
	NO EE	LEE	EE	NO EE	LEE	EE
**EE mean score**	3.5 ± 1.4	7.46 ± 1.3	14.28 ± 3.4	3.33 ± 1.7	8.33 ± 1.3	15.13 ± 3.4

**Table 4:** Emotional Eater Questionnaire (EEQ) subscale scores by emotional eating category and body mass index (BMI). Disinhibition, food preferences, and sense of guilt scores are reported for normal-weight and overweight participants classified as non-emotional eaters (NO EE), low emotional eaters (LEE), and emotional eaters (EE). Data are presented as mean ± SD. Group differences were assessed using the Kruskal–Wallis test with Dunn’s post hoc comparisons. Effect sizes (ε^2^) indicate the magnitude of between-group differences. Analyses were conducted using GraphPad Prism 10.4.

	Normal Weight Participants			Participants with Overweight			Effect Size (ε^2^ Magnitude)
	NO EE	LEE	EE	NO EE	LEE	EE	
**Disinhibition**	1.8 ± 1.09	3.98 ± 1.18	8.38 ± 2.4	1.89 ± 1.54	4.57 ± 1.48	9.6 ± 2.58	0.78
**Food** **preferences**	1 ± 0.59	2.09 ± 0.9	2.9 ± 1.26	0.78 ± 0.67	1.77 ± 1.0	2.61 ± 0.95	0.33
**Sense of guilt**	0.71 ± 0.79	1.38 ± 0.88	3 ± 1.34	2.89 ± 1.61	6.67 ± 0.88	10.69 ± 2.52	0.80

**Table 5:** Mediterranean diet adherence (MEDI-LITE score) by emotional eating category and body mass index (BMI). MEDI-LITE scores are reported for normal-weight and overweight participants classified as non-emotional eaters (NO EE), low emotional eaters (LEE), and emotional eaters (EE). Data are presented as mean ± SD. Effect sizes (ε^2^) indicate the magnitude of between-group differences.

	Normal Weight Participants			Participants with Overweight			Effect Size (ε^2^ Magnitude)
	NO EE	LEE	EE	NO EE	LEE	EE	
**MEDI LITE score**	6.37 ± 2.2	6.81 ± 2.28	7.08 ± 2.18	6.44 ± 2.88	6.47 ± 2.98	6.59 ± 2.18	0.00

**Table 6:** Spearman correlations between emotional eating variables and dietary components in normal-weight participants. Spearman correlation coefficients (r) and corresponding *p*-values are reported for associations between Emotional Eater Questionnaire (EEQ) scores, EEQ subscales, MEDI-LITE score, and individual food group consumption within the normal-weight subgroup. The strength of correlations is classified as weak or moderate according to conventional criteria.

Variables	Correlation = Coefficient (r)	*p*-Value	Interpretation
NO-EE			
**EEQ vs. fruit consumption**	−0.34	0.049	Moderate negative correlation
**EEQ vs. fish consumption**	−0.40	0.0018	Moderate negative correlation
**Sense of guilt vs. fruit consumption**	−0.38	0.025	Moderate negative correlation
**LEE**			
**EEQ vs. milk and dairy products consumption**	0.26	0.037	Weak positive correlation
**Sense of guilt vs. fruit consumption**	0.312	0.011	Moderate positive correlation
**Sense of guilt vs. extra virgin olive oil consumption**	−0.382	0.002	Moderate negative correlation
**EE**			
**EEQ vs. vegetables consumption**	0.258	0.047	Weak positive correlation
**EEQ vs. cereals consumption**	0.286	0.027	Weak positive correlation
**EEQ vs. MEDI LITE score**	0.274	0.034	Weak positive correlation
**Food preference vs. cereal** **consumption**	0.289	0.025	Weak positive correlation
**Sense of guilt vs. meat and** **derivatives**	−0.287	0.026	Weak negative correlation
**Food preference vs. extra virgin** **olive oil consumption**	−0.306	0.017	Moderate negative correlation
**Sense of guilt vs. fruit consumption**	0.392	0.002	Moderate positive correlation
**Sense of guilt vs. vegetables** **consumption**	0.398	0.002	Moderate positive correlation
**Sense of guilt vs. legumes** **consumption**	0.328	0.010	Moderate positive correlation
**Sense of guilt vs. MEDI LITE score**	0.365	0.004	Moderate positive correlation

**Table 7:** Spearman correlations between emotional eating variables and dietary components in overweight participants. Spearman correlation coefficients (r) and corresponding *p*-values are reported for associations between Emotional Eater Questionnaire (EEQ) scores, EEQ subscales, MEDI-LITE score, and individual food group consumption within the overweight subgroup. The strength of correlations is classified as weak, moderate, or strong according to conventional criteria.

Variables	Correlation Coefficient (r)	*p*-Value	Interpretation
NO-EE			
**EEQ vs. fish consumption**	0.733	0.028	Strong positive correlation
**Sense of guilt vs. fish consumption**	0.799	0.018	Strong positive correlation
**Disinhibition vs. fish consumption**	0.733	0.036	Strong positive correlation
**LEE**			
**Disinhibition vs. cereal** **consumption**	−0.397	0.0299	Moderate negative correlation
**Food preference vs. legumes** **consumption**	−0.385	0.036	Moderate negative correlation

## Data Availability

The data presented in this study are available on request from the corresponding author due to privacy reasons.

## References

[1] Berthoud H.R., Münzberg H., Morrison C.D. (2017). Blaming the Brain for Obesity: Integration of Hedonic and Homeostatic Mechanisms. Gastroenterology.

[2] Herman C.P., Polivy J. (2008). External cues in the control of food intake in humans: The sensory-normative distinction. Physiol. Behav..

[3] Gibson E.L. (2006). Emotional influences on food choice: Sensory, physiological and psychological pathways. Physiol. Behav..

[4] Devonport T.J., Nicholls W., Fullerton C. (2019). A systematic review of the association between emotions and eating behaviour in normal and overweight adult populations. J. Health Psychol..

[5] Frayn M., Knauper B. (2018). Emotional eating and weight in adults: A review. Curr. Psychol..

[6] Baik J.H. (2020). Stress and the dopaminergic reward system. Exp. Mol. Med..

[7] Russo G.L., Siani A., Fogliano V., Geleijnse J.M., Giacco R., Giampaoli S., Iacoviello L., Kromhout D., Lionetti L., Naska A. (2021). The Mediterranean diet from past to future: Key concepts from the second Ancel Keys International Seminar. Nutr. Metab. Cardiovasc. Dis..

[8] Keys A. (1975). How to Eat and Stay Well, the Mediterranean Way.

[9] Afshin A., Sur P.J., Fay K.A., Cornaby L., Ferrara G., Salama J.S., Mullany E.C., Abate K.H., Abbafati C., Abebe Z. (2019). Health effects of dietary risks in 195 countries, 1990–2017: A systematic analysis for the Global Burden of Disease Study 2017. Lancet.

[10] Swaminathan S., Dehghan M., Raj J.M., Thomas T., Rangarajan S., Jenkins D., Mony P., Mohan V., Lear S.A., Avezum A. (2021). Association of cereal grains intake with cardiovascular diseases and mortality across 21 countries in prospective urban and rural epidemiology study: Prospective cohort study. Whole grain intake and cardiovascular disease. BMJ.

[11] Obeid C.A., Gubbels J.S., Jaalouk D., Kremers S.P.J., Oenema A. (2022). Adherence to the mediterranean diet among adults in mediterranean countries: A systematic literature review. Eur. J. Nutr..

[12] Dinu M., Pagliai G., Giangrandi I., Colombini B., Toniolo L., Gensini G., Sofi F. (2021). Adherence to the Mediterranean diet among Italian adults: Results from the web.based Medi-Lite questionnaire. Int. J. Food Sci. Nutr..

[13] Monteiro C.A., Cannon G., Levy R.B., Moubarac J.C., Louzada M.L., Rauber F., Khandpur N., Cediel G., Neri D., Martinez-Steele E. (2019). Ultra-processed foods: What they are and how to identify them. Public Health Nutr..

[14] Bonaccio M., Di Castelnuovo A., Costanzo S., Ruggiero E., Esposito S., Cerletti C., Donati M.B., de Gaetano G., Iacoviello L., Bonanni A. (2025). Moli-sani study investigators. Combination of a traditional Mediterranean Diet with ultra-processed food consumption in relation to all-cause and cause-specific mortality: Prospective findings from the Moli-sani Study. Clin. Nutr..

[15] Betancourt–Nunez A., Torres–Castillo N., Martinez-Lopez E., De Loera-Rodriguez C.O., Duran-Barajas E., Marquez-Sandoval F., Bernal-Orozco M.F., Garaulet M., Vuzmanos B. (2022). Emotional eating and dietary patterns: Reflecting food choices in people with and without abdominal obesity. Nutrients.

[16] Smith J.M., Serier K.N., Belon K.E., Sebastian R.M. (2020). Evaluation of the relationships between dietary restraint, emotional eating, and intuitive eating moderated by sex. Appetite.

[17] Sofi F., Dinu M., Pagliai G., Marcucci R., Casini A. (2017). Validation of a literature-based adherence score to Mediterranean diet: The MEDI-LITE score. Int. J. Food Sci. Nutr..

[18] Garaulet M., Canteras M., Morales E., Lopez-Guimera G., Sanchez-Carracedo D., Corbalan-Tutau M.D. (2012). Validation of a questionnaire on emotional eating for use in cases of obesity: The Emotional Eater Questionnaire (EEQ). Nutr. Hosp..

[19] Konttinen H. (2020). Emotional eating and obesity in adults: The role of depression, sleep and genes. Proc. Nutr. Soc..

[20] Macht M. (2008). How emotions affect eating: A five-way model. Appetite.

[21] Van Strien T. (2018). Causes of Emotional Eating and Matched treatment of obesity. Curr. Diabetes Rep..

[22] Vasileiou V., Abbott S. (2023). Emotional eating among adults with healthy weight, overweight and obesity: A systematic review and meta-analysis. J. Hum. Nutr. Diet..

[23] Dakanalis A., Mentzelou M., Papadopoulou S.K., Papandreou D., Spanoudaki M., Vasios G.K., Pavlidou E., Mantzorou M., Giaginis C. (2023). The association of emotional eating with overweight/obesity, depression, anxiety/stress and dietary patterns: A review of the current clinical evidence. Nutrients.

[24] Madali B., Akan S.B., Ors E.D., Ayranci M., Taskin H., Kara H.H. (2021). Emotional eating behaviors during the COVID-19 pandemic: A cross–sectional study. Clin. Nutr. ESPEN.

[25] Zare H., Rahim H., Omidi A., Nernatolahi F., Sharifi N. (2024). Relationship between emotional eating and nutritional intake in adult women with overweight and obesity: A cross–sectional study. Nutr. J..

[26] Markey C.H., Strodl E., Aimè A., McCabe M., Rodgers R., Sicilia A., Lo Coco G., Dion J., Mellor D., Pietrabissa G. (2023). A survey of eating styles in eight countries: Examining restrained, emotional, intuitive eating and their correlates. Br. J. Health Psychol..

[27] Hamam M., D’Amico M., Spina D., La Via G., Di Vita G. (2024). The interplay of food-related lifestyle and eating behavior in Italian women. Front. Nutr..

[28] Muscogiuri G., Barrea L., Verde L., Docimo A., Savastano S., Di Pauli D., Colao A. (2023). Weight stigma speaks Italian, too. J. Endocrinol. Investig..

[29] Puhl R.M., Heuer C.A. (2010). Obesity stigma: Important considerations for public health. Am. J. Public Health.

[30] Carlos M., Bernabeu E., Iglesias M.T. (2020). Are adherence to the mediterranean diet, emotional eating alcohol intake and anxiety related in university students in Spain?. Nutrients.

[31] Lopez Moreno M., Garces-Rimon M., Miguel M., Iglesias Lopez M.T. (2021). Adherence to mediterranean diet, alcohol consumption and emotional eating in spanish university students. Nutrients.

[32] Hall K.D., Ayuketah A., Brychta R., Cai H., Cassimatis T., Chen K.Y., Chung S.T., Costa E., Courville A., Darcey V. (2019). Ultra-processed diets cause excess calorie intake and weight gain: An inpatient randomized controlled trial of ad libitum food intake. Cell Metab..

[33] Pagliai G., Dinu M., Madarena M.P., Bonaccio M., Iacoviello L., Sofi F. (2021). Consumption of ultra-processed foods and health status: A systematic review and meta-analysis. Br. J. Nutr..

[34] Munoz M.A., Fito M., Marrugat J., Covas M.I., Schroder H. (2009). Adherence to the mediterranean diet is associated with better mental and physical health. Br. J. Nutr..

[35] Oddo V.M., Welke L., McLeod A., Pezley L., Xia Y., Maki P., Dawn Koenig M., Kominiarek M.A., Langenecker S., Tussing-Humphreys L. (2022). Adherence to a Mediterranean Diet is associated with lower depressive symptoms among U.S. adults. Nutrients.

[36] Yau Y.H., Potenza M.N. (2013). Stress and eating behaviors. Minerva Endocrinol..

[37] Arend A.K., Blechert J., Yanagida T., Voderholzer U., Reichenberger J. (2024). Emotional food craving across the eating disorder spectrum: An ecological momentary assessment study. Eat. Weight Disord..

[38] Braden A., Kalantzis M., Dauber A., Meschino K.J., Barnhart W.R., Jordan A.K., Studer-Perez E.I. (2025). Measurement of emotional eating with the emotional eating scale, a laboratory paradigm, and ecological momentary assessment. Eat. Behav..

[39] Janek M., Kuhnova J., Cardon G., Van Dyck D., Cimler R., Elavsky S., Fezeu L.K., Oppert J.M., Buck C., Hebestreit A. (2025). Ecological momentary assessment of physical and eating behaviours: The WEALTH feasibility and optimisation study with recommendations for large-scale data collection. PLoS ONE.

[40] Borm I.M., Hartmann S., Barnow S., Pruessner L. (2025). Binge eating as emotion regulation? A meta-analysis of ecological momentary assessment studies. Clin. Psychol. Rev..

[41] Macit-Celebu M.S., Ozata-Uyar H., Yildiran H., Koksal E. (2023). Is adherence to the mediterranean diet associated with eating behaviour and emotional appetite in young women?. Rev. Esp. Nutr. Hum. Diet..

[42] Lattimore P. (2020). Mindfulness-based emotional eating awareness training: Taking the emotions out of eating. Eat. Weight Disord..

[43] Morillo-Sarto H., Lopez-del-Hoyo Y., Perez-Aranda A., Modrego-Alarcon M., Barcelo-Soler A., Borao L., Puebla-Guedea M., Demarzo M., Garcia-Campayo J., Montero-Marin J. (2022). Mindful eating for reducing emotional eating in patients with overweight or obesity in primary care settings: A randomized controlled trial. Eur. Eat. Disord. Rev..

